# Negative Pressure Wound Therapy for Residual Cavity Reduction After Open-Window Thoracostomy Following Pneumonectomy

**DOI:** 10.1016/j.atssr.2026.02.010

**Published:** 2026-02-25

**Authors:** Machiko Nishii, Satoru Okada, Takumi Nakagawa, Shunta Ishihara, Ryosuke Tokuda, Taiyo Kawamura, Masanori Shimomura, Tatsuo Furuya, Ayako Kawarazaki, Masayoshi Inoue

**Affiliations:** 1 Division of Thoracic Surgery, Department of Surgery, Graduate School of Medical Science, Kyoto Prefectural University of Medicine, Kyoto, Japan; 2 Department of Thoracic Surgery, Ayabe City Hospital, Ayabe, Japan; 3 Department of Thoracic Surgery, Japanese Red Cross Kyoto Daini Hospital, Kyoto, Japan; 4 Department of Thoracic Surgery, Japanese Red Cross Kyoto Daiichi Hospital, Kyoto, Japan; 5 Department of Thoracic Surgery, Fukuchiyama City Hospital, Fukuchiyama, Japan; 6 Department of Thoracic Surgery, Saiseikai Shiga Hospital, Ritto, Japan; 7 Division of Plastic Surgery, Department of Surgery, Graduate School of Medical Science, Kyoto Prefectural University of Medicine, Kyoto, Japan

## Abstract

Postpneumonectomy empyema with a bronchopleural fistula (BPF) usually requires open-window thoracostomy, which can leave a large difficult-to-close residual cavity when muscle or omental flaps are unavailable. An 81-year-old man who developed empyema with a BPF after completion left pneumonectomy underwent open-window thoracostomy. The BPF closed with granulation, and the large pleural cavity remained. Negative-pressure wound therapy (−50 to −75 mm Hg) achieved >50% cavity reduction within 10 days, enabling definitive thoracoplasty. Negative-pressure wound therapy appears a safe and effective option for reducing postpneumonectomy cavities and facilitating chest closure when conventional filling procedures are not feasible.

Postpneumonectomy empyema with a bronchopleural fistula (BPF) remains a severe, occasionally fatal, complication, usually requiring open-window thoracostomy (OWT) to control infection and prevent aspiration pneumonia. However, even after fistula closure, a large pleural cavity may persist, making definitive chest closure challenging. Well-vascularized tissues such as the latissimus dorsi muscle or omentum are used to obliterate dead space, but this may be unavailable for patients with previous thoracic or abdominal surgery. We applied negative-pressure wound therapy (NPWT) after bronchial stump closure, achieving a rapid cavity reduction and successful closure by thoracoplasty.

An 81-year-old man with diabetes mellitus and distal gastrectomy for gastric cancer underwent thoracoscopic left upper lobectomy for lung adenocarcinoma (pT2aN0M0, stage IB). On postoperative day (POD) 41 he developed hemoptysis owing to a bronchovascular fistula, necessitating a completion left pneumonectomy via posterolateral thoracotomy.

On POD 9 post-pneumonectomy, a BPF developed, and an OWT was performed on POD 23. Local management included daily gauze packing and systemic antibiotics. To facilitate early BPF control, a straight silicone stent was placed from the trachea to the right main bronchus; however, fistula closure was not achieved with the stent alone. Therefore, topical basic fibroblast growth factor spray and enhanced nutritional support (gradually increased to 2100 kcal/d, approximately 35 kcal/kg × ideal body weight) were introduced. The prognostic nutritional index, an immune-nutritional marker calculated as 10 × serum albumin (g/dL) + 0.005 × total lymphocyte count (cells/mm^3^) and associated with postoperative complications,^1^ was low (42) prior to left upper lobectomy and further decreased to 20 at the time of OWT. After enhanced nutritional therapy, the prognostic nutritional index gradually increased to 28 in parallel with infection control, and the fistula closed on day 81 after OWT. After fistula closure, the tracheal stent was removed. However, the residual cavity showed only minimal reduction over the next 2 months (Figures 1A, 1B).

**Figure 1:**
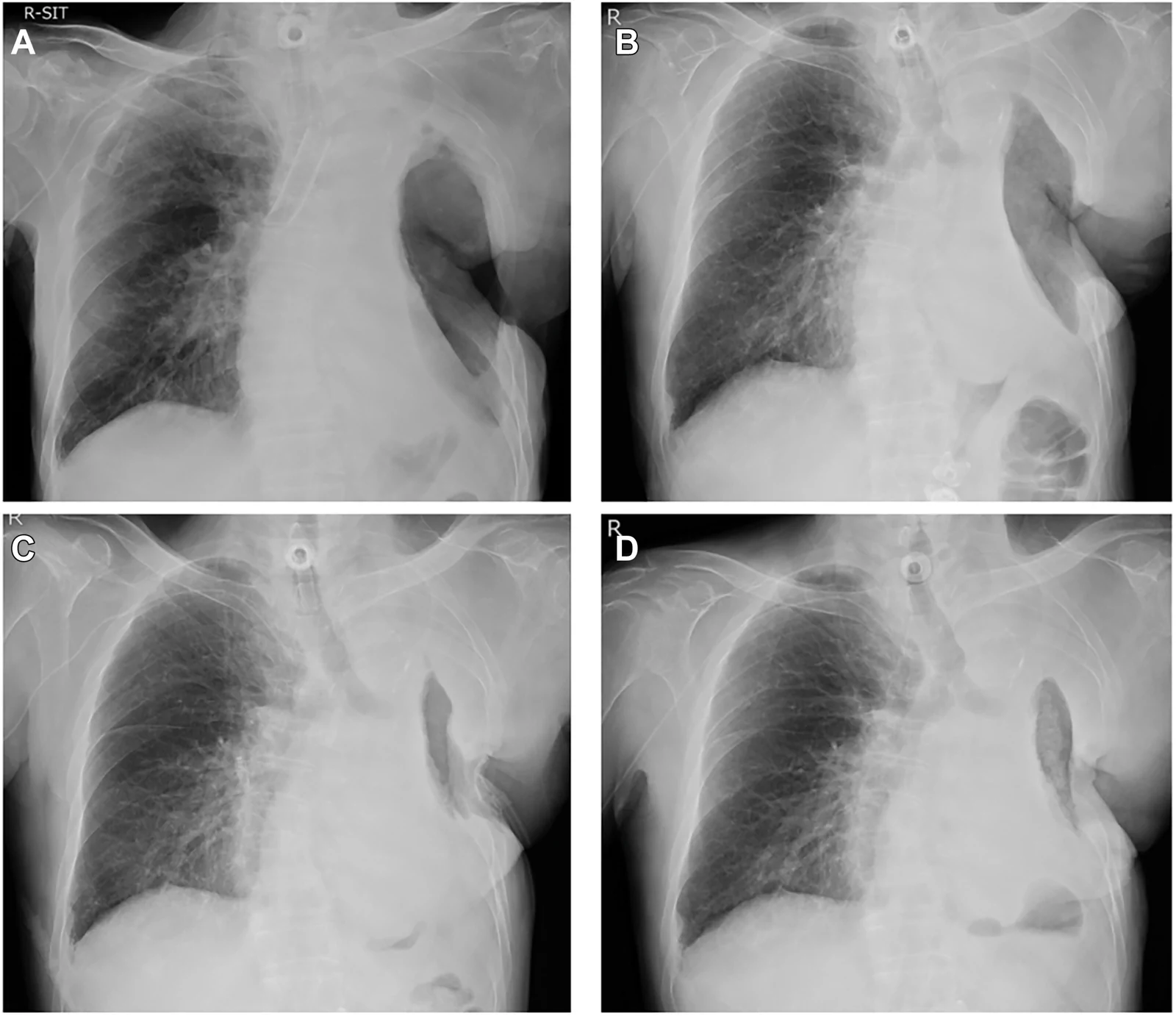
Serial chest radiographs showing progressive postpneumonectomy cavity reduction during negative-pressure wound therapy (NPWT). (A) At the time of fistula closure. (B) Two months later, pre-NPWT initiation, with only slight residual cavity reduction. (C) Day 4 of NPWT, with evident mediastinal shift and diaphragmatic elevation. (D) After NPWT completion, with residual cavity reduction <50% its original volume.

The omentum was unavailable because of prior gastrectomy and the latissimus dorsi muscle had been transected during the thoracotomy; therefore, they could not be used for space obliteration. With infection clinically controlled and the fistula closed, the intrathoracic cavity wall was covered with mature granulation tissue without exposure of major vessels or cardiac structures. NPWT was initiated using the 3M ActiV.A.C. system (Solventum), in close collaboration with the plastic surgery team for its implementation and management. A nonadherent gauze layer was placed over the pleura, and the dedicated sponge was inserted (Figure 2). Negative pressure was started at −50 mm Hg, following prior reports.^2^, ^3^, ^4^, ^5^ The initial cavity volume, measured according to saline instillation, was 250 mL. Bedside sponge dressing changes occurred twice weekly without analgesia or sedation, with intrathoracic irrigation using normal saline performed during each dressing change.

**Figure 2:**
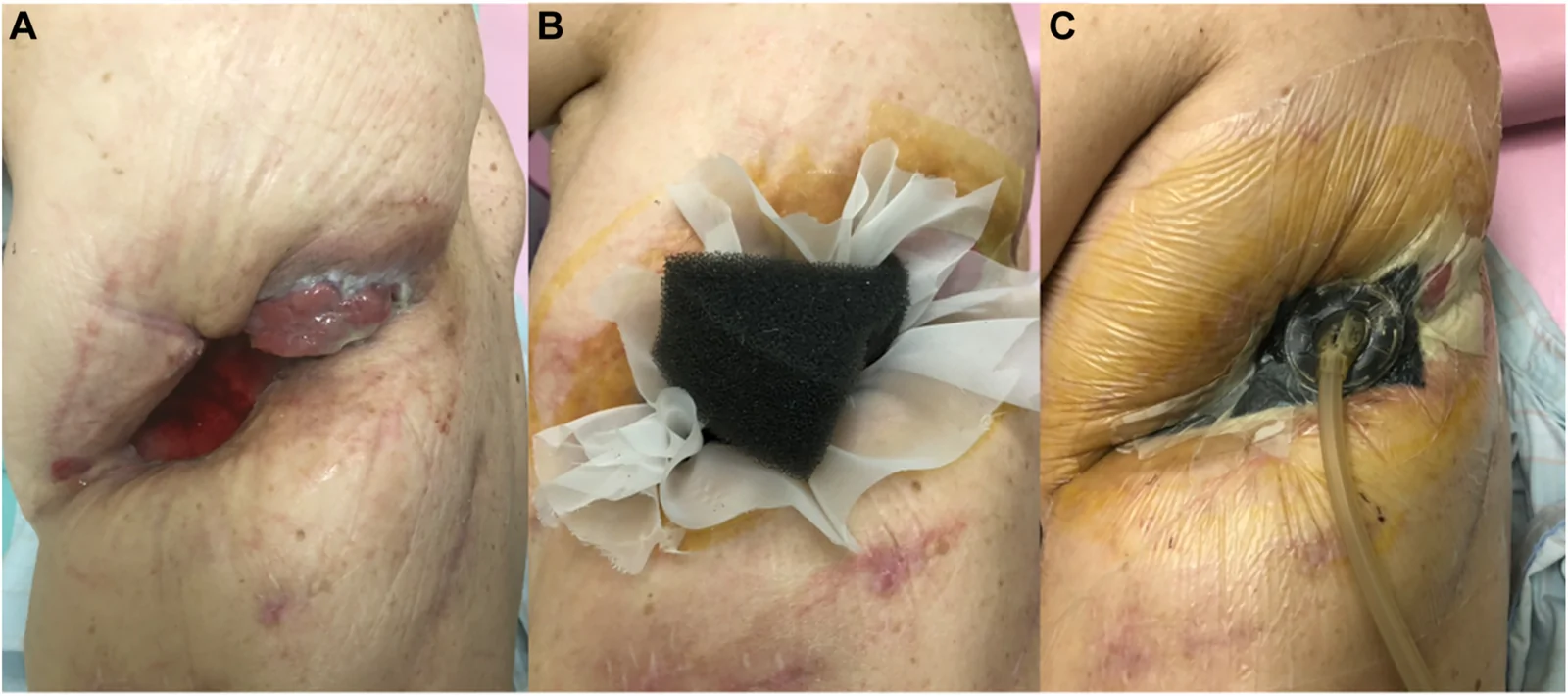
Negative-pressure wound therapy (NPWT) application. (A) Open-window thoracostomy before NPWT. (B) Nonadherent gauze and sponge placement. (C) Sealing and initiation of suction (−50 to −75 mm Hg).

By day 4 of NPWT, chest radiography showed elevation of the left hemidiaphragm and mediastinal shift, without adverse effects such as hypotension, dyspnea, or pain (Figure 1C). The pressure was then increased to −75 mm Hg. By day 10, the cavity volume had decreased to ∼100 mL (a 60% reduction). No further decrease was observed thereafter, and NPWT was discontinued after 25 days with no complications such as recurrent fistula, bleeding, or infection (Figure 1D). After NPWT, bacterial load in the pleural cultures decreased markedly (Supplemental Figure), although complete sterilization was not achieved. The reduced cavity size, along with the patient’s stable systemic and local infection status, supported the decision to proceed with definitive OWT closure. Because a small amount of residual intrathoracic bacteria was detected, intravenous antibiotics were administered starting 14 days before definitive closure.

Thoracoplasty with partial resection of the left third–seventh ribs was performed 178 days after OWT (3 days after NPWT discontinuation). During surgery, NPWT-induced granulation had thickened the pleura and intercostal muscles, which were mobilized from the ribs and inverted into the residual cavity, enabling tension-free obliteration (Supplemental Video). Drains were placed in the thoracic cavity and subcutaneous space and connected to a J-VAC suction reservoir (Johnson & Johnson). The thoracic drain was removed on POD 14, and the subcutaneous drain was removed on POD 18. The pleural cavity was completely eliminated (Figure 3). The patient has remained free of empyema, BPF, and cancer for 30 months and continues daily activities.

**Figure 3:**
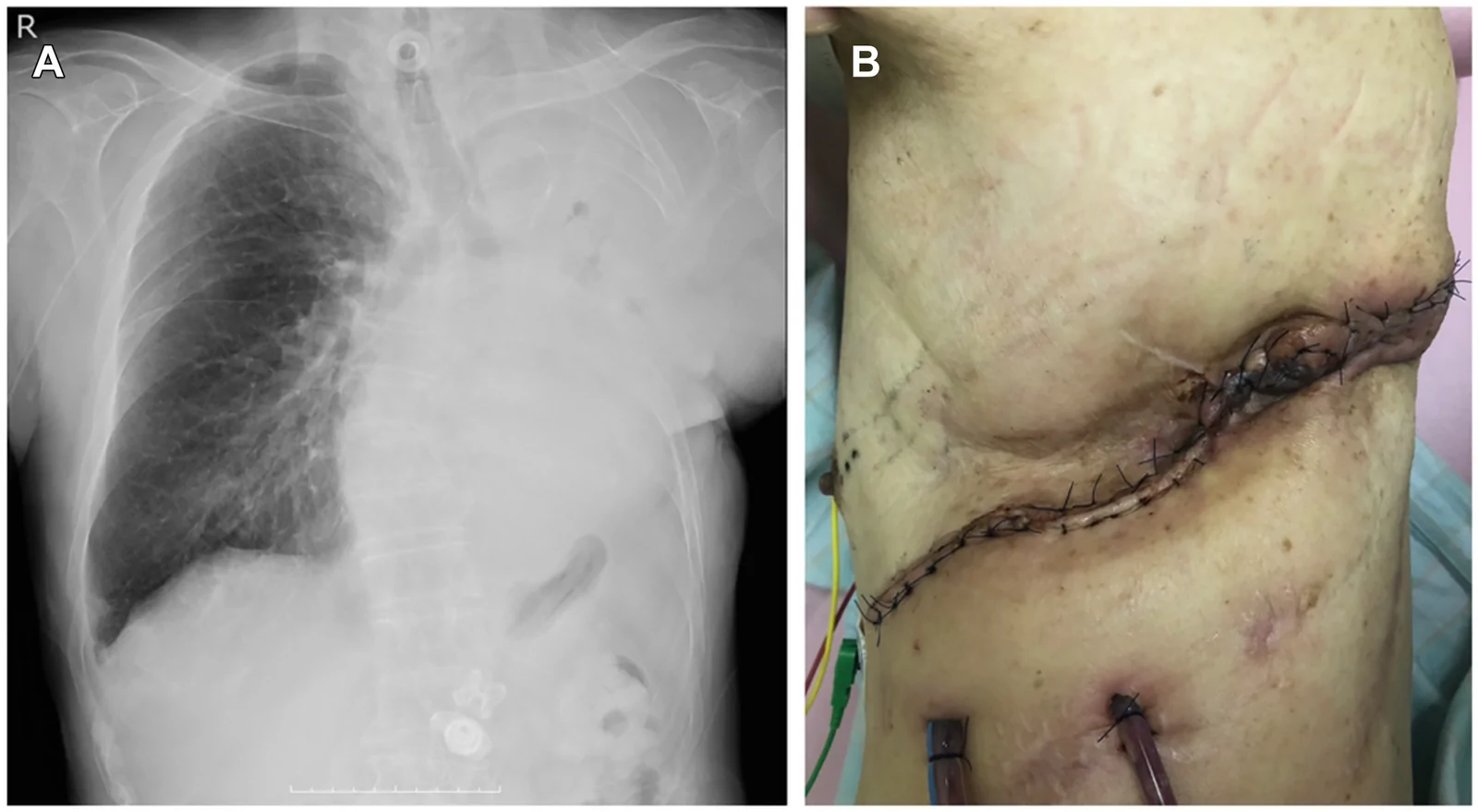
Postthoracoplasty chest radiograph (A) and (B) appearance confirming cavity obliteration.

## Comment

Postpneumonectomy empyema treatment with a BPF requires infection control, pleural cavity reduction, and secure fistula closure. NPWT is a valuable adjunct, promoting both cavity decontamination and gradual space contraction through continuous negative pressure and enhanced perfusion.

NPWT consistently demonstrates high chest closure rates (≥90%) and an excellent safety profile.^2^, ^3^, ^4^, ^5^ Perentes and associates^2^ first reported the feasibility and safety of intrathoracic NPWT after pneumonectomy, achieving marked cavity contraction without bleeding complications. Palmen and colleagues^3^ reported shorter OWT duration (median, 39 vs 933 days) and higher closure rates with NPWT compared with conventional management. When combined with bronchial closure, NPWT achieved a 90% success rate in empyema with a BPF.^4^ In our case, NPWT was initiated after BPF closure, when the cavity surface was lined with granulation tissue and no major vessels were exposed, allowing gradual negative pressures to be applied safely and effectively. The optimal level of negative pressure should be individualized for each patient, with particular caution during the initiation phase. In addition, if residual intrathoracic necrotic tissue is present before NPWT initiation, thorough debridement should be performed in advance to prevent exacerbation of infection under airtight conditions. This case underscores that NPWT can be effective even when muscle or omental flaps are unavailable, reinforcing its role as a bridging or alternative strategy for managing thoracic cavities. Although other space-obliterating options may be considered, they are generally more invasive and may compromise postoperative quality of life compared with NPWT.

The mechanism of cavity shrinkage likely involves multiple factors. Granulation tissue formation represents the expected effect of NPWT.^6^ Moreover, mechanical retraction of the chest wall, diaphragmatic elevation, and mediastinal shift are specific to its intrathoracic application. These effects work synergistically to promote cavity collapse and stabilization, thereby facilitating subsequent thoracoplasty.

The pleural cultures were never completely sterile; however, prior reports^2^^,^^5^ indicate that complete bacterial eradication is not always essential if the cavity is lined with vascularized granulation tissue and the infection is clinically controlled. Our case further suggests that even in the absence of omental filling, long-term control of empyema can be achieved when the residual cavity is completely obliterated through careful surgical manipulation after NPWT.

In conclusion, this case highlights the practical value of NPWT as a bridging therapy for patients who lack feasible filling materials. Through safely reducing cavity size and bacterial burden, NPWT can simplify definitive closure and improve outcomes in complex postpneumonectomy empyema.
