# Supplementary material for: Negative Pressure Wound Therapy for Residual Cavity Reduction After Open-Window Thoracostomy Following Pneumonectomy
Source: Ann Thorac Surg Short Rep. 2026 Feb 25;4(3):952–6. doi: 10.1016/j.atssr.2026.02.010 (PMC13555435; doi:10.1016/j.atssr.2026.02.010)
Supplement: Supplementary file 1 [file mmc1.pdf]

8 days after OWT		Before NPWT		After NPWT	
Klebsiella pneumoniae	2+	Klebsiella pneumoniae	1+	E.coli	1+
E.coli	2+	E.coli	3+	Serratia marcescens	1+
Enterococcus faecium	2+	Serratia marcescens	3+	Corynebacterium striatum	2+
Enterococcus faecalis	2+	Corynebacterium striatum	2+		
Acinetobacter species	3+				
Candida albicans	3+				

Supplementary Figure: Transition of bacterial cultures before and after NPWT showing decreased bacterial load in the pleural cavity. Findings are shown at 8 days after OWT, before NPWT initiation, and after NPWT initiation. The bacterial load decreased over time.

NPWT, negative-pressure wound therapy; *OWT*, open-window thoracostomy.
